# Supplementary material for: The extent of nitrogen isotopic fractionation in rumen bacteria is associated with changes in rumen nitrogen metabolism
Source: PLoS One. 2023 Sep 13;18(9):e0291243. doi: 10.1371/journal.pone.0291243 (PMC10499230; doi:10.1371/journal.pone.0291243)
Supplement: S1 Table — (DOCX) [file pone.0291243.s001.docx]

**The extent of nitrogen isotopic fractionation in rumen bacteria is associated with changes in rumen nitrogen metabolism**

G. Cantalapiedra-Hijar^1#*^, G. Martinez-Fernandez^2#*^, E. Forano^3^, S. Denman^2^, D. P. Morgavi^1^, C. S. McSweeney^2^

^1^INRAE, Université Clermont Auvergne, Vetagro Sup, UMRH, 63122 Saint-Genes-Champanelle, France

^2^Agriculture and Food, CSIRO, St Lucia, QLD 4067, Australia

^3^INRAE, Université Clermont Auvergne, UMR 454 MEDIS, 63122 Saint-Genès-Champanelle, France

^#^These authors equally contributed to this study

*Corresponding authors: [gonzalo.cantalapiedra@inrae.fr](mailto:gonzalo.cantalapiedra@inrae.fr); [gonzalo.martinezfernandez@csiro.au](mailto:gonzalo.martinezfernandez@csiro.au)

**S1 Table**. Composition of in vitro media for experiment 1 and 2.

| For 1000 ml | Experiment 1 | Experiment 2 |
| --- | --- | --- |
| Clarified rumen fluid^a^ | 300 ml | 300 ml |
| Yeast extract | 0.1 g | 0.1 g |
| Cellobiose | 0.5 g | - |
| Starch | 0.5 g | - |
| Cellulose (cotton thread and acid swollen filter paper) | - | 100 mg |
| NaHCO3 | 8 g | 8 g |
| Mineral Solution (1)^b^ | 150 ml | 150 ml |
| Mineral Solution (2)^c^ | 150 ml | 150 ml |
| Pfenning Trace elements^d^ | 1 ml | 1 ml |
| VFA mix^e^ | 3.1 ml | 3.1 ml |
| Haemin | 10 ml | 10 ml |
| L-Cysteine HCL-H2O | 0.5 g | 0.5 g |
| Resazurin | 1 ml | 1 ml |
| Vitamin solution^f^ | 100 µl | 100 µl |

^a^ Obtained from cannulated sheep fed a straw diet (Exp #1) or from stomach tubing cattle grazing low quality pastures (Exp #2)

^b^ 3 g K_2_HPO_4_/l distilled H_2_O

^c^ g/l distilled H_2_O: 0.6 g CaCl_2_; 3 g KH_2_PO_4_; 6 g NaCl; 6.45 g Na_2_SO_4_; 0.124 g (NH_4_)_2_(SO_4_); 0.6 g MgSO_4_ 7H_2_O.

^d^ mg/l distilled H_2_O: 300 mg H_3_BO_3_; 100 mg ZnSO_4_7H_2_O; 30 mg MnCl_2_4H_2_O; 20 mg CoCl_2_6H_2_O; 30 mg Na_2_MoO_4_2H_2_O; 10 mg Na_2_SeO_3_; 20 mg NiCl_2_; 10 mg CuCl_2_2H_2_O; 150 mg FeCl_2_4H_2_O.

^e^ The volatile fatty acid mixture was prepared by mixing 17 ml of glacial acetic acid, 6 ml of propionic acid, 4 ml of n-butyric acid and 1 ml of each n-valeric acid, isovaleric acid, isobutyric acid and 2- methylbutyric acid. The solution was made to 1 litre of distilled H_2_O

^f^ Filter-sterilised vitamin solution was added after autoclaving. g/l distilled H_2_O: 0.2 g pyridoxine hydrochloride; 0.2 g riboflavin; 0.2 g thiamine hydrochloride; 0.2 g nicotinamide; 0.2 g DL-pantothetic acid hemicalcium salt; 0.01 g p-amino benzonic acid; 0.005 g folic acid; 0.005 g d-biotin; 0.0005 g cyanocobalamin.
