# Supplementary material for: The extent of nitrogen isotopic fractionation in rumen bacteria is associated with changes in rumen nitrogen metabolism
Source: PLoS One. 2023 Sep 13;18(9):e0291243. doi: 10.1371/journal.pone.0291243 (PMC10499230; doi:10.1371/journal.pone.0291243)
Supplement: Attachment — Submitted filename: PONE-N isotopic-Report.docx [file pone.0291243.s002.docx]

**The extent of nitrogen isotopic fractionation in rumen bacteria is associated with changes in rumen nitrogen metabolism**

Thank you for sending this manuscript to me. The manuscript discusses the effect of N sources and levels on the Δ^15^N_bacteria-substrate_ of pure and mixed bacterial rumen bacteria. The idea is brilliant and the manuscript provides more details about the N metabolism by rumen bacteria. The manuscript still needs some improvements to be published.

**Abstract:**

Could you make the experimental design clearer in the abstract?

Please write the full name of bacterial strains that were used.

**Introduction:**

Line 60: Could you explain the AA?

**Material and methods**

Why didn’t you use any component of animal diet (hay, soya meal) as substrate in the bacterial incubation besides the organic and inorganic nitrogen sources?

Why the authors used a mixture of VFAs in the growth of the pure strains, the pure strains do not need all those VFAs in the growth. Also, the authors should explain the presence of all the VFAs in the supernatant of the pure culture even after the subtraction of the quantity of VFAS of the incubation medium, because the single pure culture cannot produce all the VFAs. This point should be explained in the discussion.

**Results**

The authors should include a complete image of the effect of nitrogen sources on the alpha diversity and relative abundance of the bacterial community in mixed culture.

Where is the data on gas production and gas fraction besides the changes in the archaeal community in the mixed culture? This data could explain the relationship between nitrogen sources and methane and other gas production.

**Discussion**

The effect of nitrogen sources on the diversity and relative abundance of microbial communities should be discussed.

The authors should justify the presence of mixed VFAs in the culture of single strains as the single strain produces one or more VFAs.

It is not necessary to repeat the sentence “previously named *Prevotella ruminicola*”.
